# *Mycobacterium marinum* Hand Infection in a Kidney Transplant Recipient: A Diagnostic Challenge

**DOI:** 10.3390/idr18050095

**Published:** 2026-08-29

**Authors:** Mariantonia Braile, Giusy Corvino, Rosamaria Abate, Mariano Conticelli

**Affiliations:** UOSD Laboratorio Territoriale SS Annunziata, ASL Napoli 1, 80145 Naples, Italy

**Keywords:** *Mycobacterium marinum*, nontuberculous mycobacteria, aquarium infection, kidney transplantation, immunosuppression, hand infection, granuloma, PCR

## Abstract

Background: *Mycobacterium marinum* is a slow-growing nontuberculous mycobacterium associated with aquatic environments and may cause chronic skin and soft-tissue infections following minor skin trauma. Diagnosis can be delayed because clinical manifestations may mimic conventional bacterial infections. We report an unusual case in a kidney transplant recipient with a positive QuantiFERON-TB Gold test and subsequent *M. marinum* infection of the hand. Case presentation: A 53-year-old man with a history of kidney transplantation and long-term immunosuppressive therapy developed progressive swelling, pain, erythema, and functional impairment of the right third finger. After independently discontinuing tacrolimus, mycophenolate mofetil, and prednisone following a positive QuantiFERON-TB Gold test, he developed a progressive hand infection despite empirical ciprofloxacin therapy. He reported repeated exposure to a domestic freshwater aquarium and frequent contact with aquarium water and filtration equipment, with minor skin abrasions. Surgical exploration revealed dense, whitish, caseous-appearing material. Histopathology demonstrated chronic granulomatous inflammation, while Ziehl–Neelsen staining showed acid-fast bacilli. Culture yielded slow-growing photochromogenic colonies at 28–32 °C, and species-specific PCR confirmed *M. marinum*. No antimicrobial susceptibility testing was performed. Following surgical drainage and six days of empirical ciprofloxacin, no targeted antimycobacterial therapy was administered. The patient achieved complete clinical resolution, and immunosuppressive therapy was gradually reintroduced approximately four weeks after surgery. Conclusions: This case highlights the importance of considering *M. marinum* in persistent or treatment-refractory hand infections, particularly in patients with aquarium exposure and impaired immunity. Early tissue sampling, appropriate culture conditions, and molecular identification are essential for diagnosis. A positive QuantiFERON-TB Gold result should be interpreted cautiously because cross-reactivity with *M. marinum* is possible. The favorable outcome observed after surgical source control and a short empirical course of ciprofloxacin is unusual and should not be interpreted as evidence supporting short-course monotherapy for deep *M. marinum* infection. Because antimicrobial susceptibility testing and serial follow-up cultures were unavailable, the contribution of ciprofloxacin to microbiological clearance cannot be determined.

## 1. Introduction and Clinical Significance

*Mycobacterium (M.) marinum* is a slow-growing environmental mycobacterium belonging to the group of nontuberculous mycobacteria (NTM). *M. marinum* is widely distributed in freshwater and saltwater environments and has been isolated from fish, aquatic plants, biofilms, aquaria, and aquarium equipment [1,2]. Because its optimal growth temperature is approximately 28–32 °C, human infection predominantly affects relatively cooler anatomical sites, particularly the distal upper extremities [1,2].

Human infection usually follows direct inoculation of contaminated water into minor skin injuries sustained during occupational or recreational aquatic activities. Historically referred to as “swimming pool granuloma,” *M. marinum* infection is now more frequently associated with domestic aquarium maintenance, fish handling, aquaculture, and exposure to marine animals [1,2].

Clinical manifestations are often nonspecific and may resemble bacterial cellulitis, pyogenic abscesses, sporotrichosis, foreign-body granulomas, fungal infections, or inflammatory arthropathies. Consequently, diagnosis may be delayed for several weeks or months [2,3]. In immunocompetent individuals, infection is generally localized to the skin and subcutaneous tissues. In contrast, immunocompromised patients—including solid-organ transplant recipients and individuals receiving corticosteroids or other immunosuppressive therapies—may develop deeper soft-tissue involvement, tenosynovitis, septic arthritis, osteomyelitis, or disseminated disease [2,3].

Diagnosis may be challenging because routine bacterial cultures are frequently negative and mycobacterial cultures require specific incubation temperatures and prolonged incubation periods. Histopathological examination may demonstrate granulomatous inflammation, sometimes with necrosis, whereas Ziehl–Neelsen (ZN) staining may have limited sensitivity because the number of acid-fast bacilli in tissue specimens can be low. Molecular methods, including polymerase chain reaction (PCR)-based assays, can facilitate more rapid species-level identification once an NTM has been isolated [2,3,4,5].

The optimal treatment of *M. marinum* infection has not been standardized because of the rarity of the disease and the absence of randomized controlled trials. Management generally involves prolonged antimicrobial therapy tailored to the anatomical extent of infection and the patient’s immune status, with surgical debridement or drainage when deep soft-tissue infection, abscess formation, tenosynovitis, or extensive necrosis is present [2,3,4,5]. In immunocompromised patients, treatment may be more complex and may require prolonged therapy and close clinical follow-up, particularly when deeper or disseminated infection is present [2,3,4,5]. The duration and composition of therapy are particularly uncertain in deep infections, for which published experience is based largely on case reports, case series, and retrospective studies.

Here, we describe a microbiologically confirmed case of *M. marinum* infection involving the third finger of the dominant hand in a kidney transplant recipient with repeated exposure during cleaning of a domestic tropical freshwater aquarium. The diagnosis was established following surgical drainage of a lesion initially suspected to represent a conventional bacterial infection. The unusual clinical resolution after surgical source control and only six days of empirical ciprofloxacin, without subsequent targeted antimycobacterial therapy, provides an opportunity to discuss the diagnostic challenges and the uncertainty surrounding treatment response in this setting.

## 2. Case Presentation

### 2.1. Patient Information and Medical History

A 53-year-old man presented with progressive pain, swelling, and functional impairment of the third finger of the right hand. His medical history was significant for end-stage renal disease treated with kidney transplantation and severe psoriatic arthritis requiring long-term immunosuppressive therapy. Kidney transplantation had been performed in 2020. Before discontinuation, his maintenance immunosuppressive regimen consisted of tacrolimus 2 mg twice daily (target trough concentration, 4–6 ng/mL), mycophenolate mofetil 500 mg twice daily, and prednisone 5 mg once daily [6]. The patient had been maintained on chronic immunosuppressive therapy following transplantation. However, on 12 September 2023, he independently discontinued all immunosuppressive medications after receiving a positive QuantiFERON-TB Gold test during routine screening. The decision was not physician-directed, and the patient had not undergone specialist assessment before discontinuing treatment. He had interpreted the positive result as evidence of active tuberculosis. Subsequent evaluation for possible tuberculosis included chest imaging, which showed no radiological findings suggestive of active pulmonary tuberculosis. The patient reported no symptoms suggestive of active tuberculosis, including persistent cough, fever, night sweats, hemoptysis, or unexplained weight loss. Based on the available clinical and radiological evaluation, no evidence of active tuberculosis was identified. The positive QuantiFERON-TB Gold result was therefore interpreted in the context of possible *M. tuberculosis* infection rather than as evidence of active disease. IGRAs cannot distinguish latent tuberculosis infection from active tuberculosis and should be interpreted together with clinical, radiological, and, when indicated, microbiological findings [7]. Importantly, some NTM species, including *M. marinum* and *M. kansasii*, possess ESAT-6 and CFP-10 antigens that may cross-react with the antigens used in IGRAs [8]. Thus, cross-reactivity provides a biologically plausible explanation for the positive QuantiFERON-TB Gold result in this patient, but this remains speculative. The subsequent identification of *M. marinum* does not establish that the positive IGRA resulted from cross-reactivity, and concomitant *M. tuberculosis* infection cannot be excluded solely on this basis. No known history of recent travel, tuberculosis exposure, or occupational risk factors for tuberculosis or other infectious diseases was reported.

### 2.2. Immunological Assessment

Before discontinuation of immunosuppressive therapy, the patient’s peripheral lymphocyte count and T-cell subsets were within the available laboratory reference ranges, and serum immunoglobulin concentrations were broadly preserved. Tacrolimus exposure was monitored by trough concentration before treatment discontinuation and subsequently declined following interruption of therapy [9]. These findings were considered descriptive of the patient’s peripheral immune profile and were not interpreted as evidence of functional immune recovery. Peripheral lymphocyte counts and T-cell subset enumeration provide quantitative information regarding circulating immune cells but cannot, in isolation, establish restoration of pathogen-specific cellular immunity [10]. Therefore, the available laboratory data do not allow a reliable assessment of whether withdrawal of immunosuppressive therapy resulted in clinically meaningful functional immune recovery.

### 2.3. Environmental and Exposure History

Upon detailed anamnesis, the patient reported repeated exposure to a domestic tropical freshwater aquarium maintained at home. Specifically, he performed frequent cleaning of the aquarium filtration system, an activity associated with direct contact with aquarium water, organic debris, and contaminated aquarium equipment. During these maintenance procedures, the patient reported multiple minor skin abrasions involving the hands, although no single major traumatic event, animal bite, or puncture injury was identified. No personal protective equipment such as gloves was used during aquarium handling.

Following diagnosis, the patient was advised to avoid direct contact between aquarium water and broken skin and to use waterproof protective gloves when cleaning the aquarium or handling the filtration system. He was also advised to avoid aquarium maintenance when open cuts or skin abrasions were present. These measures are consistent with published preventive recommendations for individuals with regular aquarium exposure, particularly those with skin lesions or abrasions [11,12]. At the three-month follow-up, the patient reported adherence to these preventive measures, including regular use of waterproof protective gloves during aquarium maintenance. No recurrence of hand lesions or other clinically evident *M. marinum*-related infection was observed during follow-up [12,13].

### 2.4. Initial Symptom Development and Disease Progression

The patient initially noted localized swelling and mild discomfort of the distal phalanx of the right third finger. At the early stage, symptoms were nonspecific and were interpreted as a possible minor soft-tissue injury. Over the following days, however, the lesion progressively worsened, with increasing swelling, erythema, and tenderness.

An empirical course of oral ciprofloxacin was prescribed in the community setting because the clinical presentation was initially considered compatible with a bacterial soft-tissue infection or early abscess. At that time, there was no specific clinical suspicion of *M. marinum* or other NTM infection. Despite empirical antibiotic therapy, the clinical condition failed to improve, and the lesion progressively evolved with increasing local inflammation and functional impairment. The lack of clinical response to empirical ciprofloxacin did not by itself establish an NTM infection but contributed, together with the chronic and atypical clinical course, to the subsequent consideration of an alternative infectious etiology.

### 2.5. Emergency Department and Surgical Specialist Evaluation

On 1 October 2023, the patient underwent surgical hand specialist assessment at the Emergency Department of “Ospedale dei Pellegrini” (Naples, Italy).

Clinical examination revealed:

▪ Diffuse fusiform swelling of the third ray of the right hand;
▪ Pronounced erythema involving the distal and middle phalanges;
▪ Localized warmth and tenderness on palpation;
▪ Severe pain limiting both active and passive range of motion;
▪ Functional impairment of the affected finger;
▪ Presence of a small cutaneous fistula with spontaneous drainage of whitish material.

Despite the significant local inflammatory findings, the patient remained afebrile and hemodynamically stable, with no signs of systemic infection or sepsis. No regional lymphadenopathy or proximal lymphangitic spread was observed at the time of evaluation. The combination of fusiform swelling, pain with passive movement, marked tenderness, and functional impairment raised clinical suspicion for a deep hand infection with possible flexor tendon sheath involvement, consistent with features described in the clinical assessment of flexor tenosynovitis [3,14]. Although these findings are suggestive, they are not specific for pyogenic flexor tenosynovitis and may also occur in other infectious or inflammatory conditions [3,15]. A representative image of the clinical presentation is shown in Figure 1.

### 2.6. Laboratory and Preoperative Assessment

Initial laboratory investigations showed moderate inflammatory abnormalities consistent with a localized infectious or inflammatory process. Renal function showed a mild deterioration compared with the patient’s baseline. These laboratory findings were nonspecific and could not distinguish between infectious and non-infectious inflammation [16]. The patient’s immunological profile had been assessed before discontinuation of immunosuppressive therapy, as described above; however, these data were considered insufficient to assess functional immune recovery. Routine microbiological evaluation included Gram staining and bacterial cultures. Given the lack of response to empirical antibacterial therapy, the progressive course of the lesion, and the atypical clinical presentation, urgent surgical intervention was performed for both therapeutic decompression and diagnostic clarification. Surgical exploration allowed source control and collection of tissue specimens for microbiological and histopathological examination, which are particularly important in suspected deep or atypical hand infections [14,17].

### 2.7. Surgical Intervention and Intraoperative Findings

After informed consent, surgical exploration was performed under local anesthesia.

A longitudinal incision and fasciotomy of the third finger were performed. Upon exploration of the affected soft tissues, a substantial amount of dense, whitish, poorly liquefied material with a caseous appearance was evacuated. The intraoperative findings were notable for:

▪ Abundant dense, whitish, caseous-appearing material with limited liquefaction;
▪ Only a limited amount of conventional purulent exudate;
▪ Diffuse involvement of the surrounding subcutaneous soft tissues rather than a clearly circumscribed fluid collection;
▪ No gross bone exposure or macroscopic evidence of osseous destruction at the time of surgery.

The macroscopic appearance was considered atypical for an acute pyogenic abscess because the material was predominantly dense, whitish, and poorly liquefied rather than consisting of the liquid or creamy purulent exudate typically encountered in an acute bacterial abscess. No foul-smelling purulent material or well-demarcated cavity containing liquefied pus was observed. The thick, granular, caseous-appearing material, together with the relatively chronic clinical course and the absence of a typical well-formed purulent collection, raised consideration of an atypical infectious process, including granulomatous or mycobacterial infection [14,18].

Given the atypical intraoperative appearance, the patient’s history of repeated aquarium exposure, and the lack of response to empirical antibacterial therapy, mycobacterial infection was included in the differential diagnosis and dedicated mycobacterial investigations were therefore performed [14,17].

Two independent tissue samples were obtained from the lesion for comprehensive microbiological analysis, including:

▪ Aerobic and anaerobic bacterial cultures;
▪ Mycobacterial culture;
▪ Cytological examination;
▪ Molecular diagnostic testing.

Obtaining tissue specimens during surgical exploration allowed simultaneous therapeutic management and microbiological investigation, which is particularly important in atypical or chronic hand infections where routine bacterial cultures may be insufficient [14,17]. The surgical field was extensively irrigated with sterile saline solution. The wound was left partially open to facilitate postoperative drainage and to avoid closure under tension, in accordance with principles used in the surgical management of deep hand infections [14,17].

### 2.8. Histopathological and Cytological Findings

Histopathological examination demonstrated chronic granulomatous inflammation with focal necrosis and a mixed inflammatory infiltrate, including epithelioid histiocytes and occasional multinucleated giant cells. No malignancy was identified. These findings were compatible with a chronic granulomatous infectious process but were not specific for *M. marinum* [19,20]. Cytological examination showed necrotic and granular material with macrophages and mixed inflammatory cells, consistent with a chronic inflammatory/infectious process but without species-level diagnostic specificity. The absence of morphologically identifiable mycobacteria did not exclude *M. marinum* infection, as acid-fast bacilli may be sparse or absent in tissue sections [19,20]. The diagnosis was therefore established by integrating the clinical and epidemiological context with ZN staining, mycobacterial culture, and species-specific molecular identification.

### 2.9. Microbiological and Molecular Findings

Routine Gram staining of the surgical specimens did not reveal bacterial pathogens, and routine aerobic and anaerobic cultures remained negative. ZN staining of the surgical material demonstrated scattered acid-fast bacilli, supporting the presence of an acid-fast bacillary infection but not permitting species-level identification [20,21]. Representative acid-fast bacilli identified by ZN staining are shown in Figure 2.

For mycobacterial isolation, the surgical specimens were inoculated onto Löwenstein–Jensen medium and incubated at 28–32 °C under appropriate conditions. After approximately 40 days of incubation, slow-growing colonies became visible on the culture medium. The prolonged incubation period and preferential growth at temperatures around 30 °C were compatible with the known growth characteristics of *M. marinum* [21,22].

#### 2.9.1. Identification of the Isolate

The colonies were characterized as a slow-growing NTM based on their growth characteristics, colony morphology, and pigmentation pattern.

The colonies were smooth to slightly rough, raised, and initially non-pigmented when examined without light exposure. Following exposure to light, the colonies developed yellow–orange pigmentation, consistent with a photochromogenic phenotype. This photochromogenic phenotype is characteristic of *M. marinum,* although it is not by itself sufficient for definitive species identification because other NTM may also exhibit photochromogenicity [21,22]. The characteristic photochromogenic appearance of the colonies is shown in Figure 3.

Growth was observed at 28–32 °C, whereas growth at 37 °C was absent or markedly reduced. These phenotypic characteristics were considered compatible with a photochromogenic NTM, particularly *M. marinum*, but were not regarded as sufficient for species-level identification. Species identification was subsequently confirmed by a species-specific PCR assay performed on the cultured isolate, which yielded a positive result for *M. marinum*. Molecular identification provided species-level confirmation of the isolate and, together with the culture and phenotypic findings, established the microbiological diagnosis [21,22]. The combination of acid-fast microscopy, characteristic culture morphology and pigmentation, species-specific molecular identification, and the compatible clinical and epidemiological context established the diagnosis of *M. marinum* infection.

#### 2.9.2. Antimicrobial Susceptibility Testing

Antimicrobial susceptibility testing was not performed on the isolate according to the laboratory’s routine diagnostic protocol. Therefore, no phenotypic susceptibility profile was available to guide antimicrobial selection or to determine whether the isolate was susceptible to ciprofloxacin. This limitation is particularly relevant in the present case because clinical resolution occurred despite the absence of subsequent targeted antimycobacterial therapy. Consequently, the contribution of ciprofloxacin to microbiological clearance cannot be established [22].

### 2.10. Postoperative Management

Following surgical drainage, empirical antimicrobial therapy with ciprofloxacin 500 mg orally twice daily was continued for a total of 6 days. At the time of treatment initiation, the working diagnosis was a conventional bacterial soft-tissue infection, and the microbiological diagnosis of *M. marinum* was not yet available. Ciprofloxacin was therefore administered empirically and was not intended as targeted therapy for *M. marinum.* After microbiological identification of *M. marinum*, no additional antimycobacterial therapy was initiated because the patient had already completed the empirical antibiotic course and was showing clear clinical improvement. In retrospect, six days of ciprofloxacin monotherapy would not be considered an adequate standard treatment for confirmed deep *M. marinum* infection. Published clinical experience generally supports prolonged antimicrobial therapy for infections involving deeper structures, whereas shorter or single-agent regimens have mainly been reported in selected superficial infections. The subsequent favorable clinical course was therefore considered unusual and should not be interpreted as evidence supporting short-course ciprofloxacin monotherapy. Surgical source control, removal of infected and inflammatory material, local wound care, and the absence of documented osteomyelitis or another deep structural focus may all have contributed to recovery. A contribution from ciprofloxacin cannot be excluded, but cannot be established because antimicrobial susceptibility testing was not performed and follow-up mycobacterial cultures were unavailable. Postoperative local wound management included sterile dressing changes every 3 days and local application of povidone–iodine according to the treating team’s protocol. The patient was monitored clinically for recurrent inflammation, wound deterioration, secondary bacterial infection, and functional impairment. A computed tomography (CT) examination of the affected finger was performed approximately 48 h after surgery to evaluate possible deeper extension of the infection. The CT scan showed diffuse soft-tissue swelling without evidence of cortical bone destruction, osteomyelitis, or a deep organized fluid collection. No gross involvement of the flexor tendon sheath was identified. Given the patient’s history of kidney transplantation and recent withdrawal of maintenance immunosuppressive therapy, immunosuppressive treatment remained discontinued during the initial postoperative period under close nephrological supervision. Approximately four weeks after surgery, following clear clinical improvement, complete resolution of local inflammatory signs, and stable renal graft function, immunosuppressive therapy was gradually reintroduced under the supervision of the nephrology and transplant teams [23]. Tacrolimus was initially restarted at a reduced dose, followed by gradual reintroduction of the remaining maintenance immunosuppressive regimen according to the patient’s clinical status. At approximately 40 days after surgery, the surgical wound was completely healed, with resolution of local swelling and erythema and restoration of active and passive finger movement. Repeat ZN staining of wound material was negative for acid-fast bacilli. Because follow-up mycobacterial cultures were not available, this result was not considered evidence of definitive microbiological eradication.

### 2.11. Follow-Up and Outcome

Clinical follow-up was performed in the outpatient setting with serial wound assessments and evaluation of functional recovery. At approximately 40 days after surgical intervention, the surgical wound was completely healed, local inflammatory signs had resolved, and active and passive finger movement had been restored. Repeat ZN staining of wound material was negative for acid-fast bacilli. This finding was consistent with clinical improvement but did not establish microbiological eradication because follow-up mycobacterial cultures were not available. At the three-month follow-up, the patient remained clinically well, with no recurrence of local inflammation, swelling, drainage, or functional impairment. He reported adherence to preventive measures during aquarium maintenance, including the use of waterproof protective gloves. No further clinically evident episode of *M. marinum* infection was observed during the available follow-up period.

## 3. Discussion

*M. marinum* is a slow-growing, photochromogenic NTM associated with freshwater and marine environments and is an established cause of skin and soft-tissue infections following aquatic exposure. Although infection is most commonly localized to the skin and subcutaneous tissues, deeper involvement of tendon sheaths, joints, and bone may occur, particularly in patients with delayed diagnosis or impaired cellular immunity [1,2,20]. The present case illustrates several clinically relevant aspects of *M. marinum* infection. First, the diagnosis should be considered in patients presenting with persistent, atypical, or treatment-refractory hand infections when there is a history of aquarium or aquatic exposure, even in the absence of a clearly recalled traumatic inoculation event [1,2,11,12]. Second, immunosuppression may increase the risk of deeper tissue involvement and a more complicated clinical course [1,2,20,24]. Third, diagnosis depends on appropriate specimen collection and communication with the microbiology laboratory because *M. marinum* grows preferentially at temperatures lower than those routinely used for many other bacterial pathogens and may therefore be missed if specific culture conditions are not requested [1,2,20,25].

### 3.1. Clinical and Epidemiological Relevance

The patient’s repeated exposure to a domestic tropical freshwater aquarium, particularly during cleaning of the filtration system, represents a highly compatible epidemiological context for *M. marinum* infection. Aquarium water, biofilms, filtration systems, and aquarium-associated materials may harbor environmental NTM, and direct contact with contaminated water or equipment may lead to inoculation through minor disruptions of the skin barrier [11,12,13,22]. Minor abrasions may provide an entry site even when the patient does not recall a specific traumatic event, a circumstance frequently reported in *M. marinum* infection [1,2,11,12]. The initial clinical presentation was nonspecific and was initially considered compatible with a conventional bacterial soft-tissue infection. Failure to respond to empirical antibacterial therapy, progressive swelling, functional impairment, and the presence of dense whitish material at surgery subsequently raised suspicion of an atypical infectious process. Such diagnostic difficulty is well described in *M. marinum* infection, which may mimic pyogenic bacterial infection, fungal disease, sporotrichosis, inflammatory disorders, or foreign-body reactions [1,2,20]. The involvement of the hand is clinically important because *M. marinum* infection may extend from superficial structures to deeper tissues, including tendon sheaths, joints, bursae, and bone. Deeper infection has been reported in approximately 20–40% of cases and is particularly relevant in patients with delayed diagnosis or impaired host immunity [1,2,20]. In immunocompromised patients, including solid-organ transplant recipients, infection may be more extensive and may occasionally disseminate [20,26]. The present case therefore reinforces the importance of obtaining a detailed environmental history in patients with chronic or atypical hand infections. In particular, aquarium maintenance should be specifically queried because patients may not spontaneously recognize this exposure as clinically relevant [1,2,12,13].

### 3.2. Diagnostic Challenges

The microbiological findings in this case demonstrate the importance of obtaining tissue specimens for mycobacterial investigation when an atypical hand infection is encountered. Routine Gram staining and aerobic and anaerobic cultures were negative, whereas ZN staining demonstrated acid-fast bacilli, supporting the presence of an acid-fast bacterial infection but not permitting species-level identification [20,25]. The isolation of a slow-growing, photochromogenic organism at 28–32 °C provided an important phenotypic clue toward *M. marinum.* Because the organism grows preferentially at lower temperatures and may require prolonged incubation, failure to communicate clinical suspicion to the microbiology laboratory may result in missed or delayed diagnosis [1,2,20,25]. In the present case, species-specific PCR subsequently confirmed *M. marinum.* Histopathology may provide supportive but nonspecific findings, including granulomatous inflammation, epithelioid histiocytes, multinucleated giant cells, and focal necrosis, while acid-fast bacilli may be sparse or absent [19,20]. Accordingly, culture and molecular identification remain essential for species-level confirmation.

### 3.3. Interpretation of the Positive QuantiFERON-TB Gold Result

An additional clinically relevant feature of this case was the positive QuantiFERON-TB Gold result obtained shortly before presentation. Interferon-gamma release assays (IGRAs) detect T-cell responses to antigens including ESAT-6 and CFP-10 and are useful for identifying immunological sensitization to *M. tuberculosis*. However, a positive result does not distinguish latent from active tuberculosis and must be interpreted in conjunction with the clinical and epidemiological context [7]. Selected NTM, including *M. marinum* and *M. kansasii*, possess homologous antigens that may result in cross-reactive interferon-gamma responses [8]. Thus, *M. marinum* infection represents a biologically plausible explanation for a positive IGRA in this patient. However, this possibility should not be interpreted as causal evidence. The subsequent microbiological confirmation of *M. marinum* does not establish that the positive QuantiFERON-TB Gold result was caused by cross-reactivity, and concomitant *M. tuberculosis* infection cannot be excluded solely on the available data. The clinically important issue in this case was the patient’s interpretation of the positive IGRA as evidence of active tuberculosis, which led to unsupervised discontinuation of maintenance immunosuppression. The subsequent evaluation did not demonstrate active pulmonary tuberculosis. This case therefore emphasizes that an isolated positive IGRA should not be interpreted as evidence of active disease or used as the sole basis for modifying immunosuppressive therapy.

### 3.4. Immunosuppression and Clinical Outcome

The patient had been receiving long-term immunosuppressive therapy following kidney transplantation. Cellular immunity, particularly T-cell-mediated responses, plays an important role in host defense against intracellular pathogens, and transplant recipients are at increased risk of opportunistic infections as a consequence of their overall net state of immunosuppression [26,27]. In this case, the patient independently discontinued tacrolimus, mycophenolate mofetil, and prednisone before presentation. Although this temporal relationship is noteworthy, the available data do not demonstrate that withdrawal of immunosuppression resulted in functional immune recovery or contributed directly to the favorable outcome. Peripheral lymphocyte and T-cell subset measurements provide quantitative information but cannot establish pathogen-specific immune competence [10]. Several factors may instead have contributed to recovery, including prompt surgical source control, removal of infected and inflammatory material, local wound care, and the absence of documented osteomyelitis or another deep structural focus. The contribution of changes in the patient’s immune status therefore remains uncertain and should be regarded as hypothesis-generating rather than causal. The case nevertheless highlights the importance of careful management of immunosuppressive therapy in transplant recipients with suspected or confirmed infection. Reduction or modification of immunosuppression may sometimes be considered during significant infection, but excessive reduction may increase the risk of graft rejection and graft dysfunction [26,27]. Decisions regarding temporary modification and subsequent reintroduction of maintenance immunosuppression should therefore be individualized and undertaken within a multidisciplinary transplant team.

### 3.5. Treatment Considerations

The treatment of *M. marinum* infection is not standardized because controlled clinical trials are lacking and the available evidence is derived predominantly from observational studies, case series, case reports, and expert opinion [1,2,5,23]. Treatment selection depends on the anatomical extent of infection, severity of disease, host immune status, potential drug interactions, and involvement of deep structures such as tendon sheaths, joints, or bone [1,2,5]. Superficial cutaneous infections may respond to a single active antimicrobial agent in selected patients, whereas deeper infections generally require prolonged treatment and are often managed with combination antimicrobial regimens [1,2,5,23]. Surgical management is particularly important when abscess formation, extensive necrosis, tenosynovitis, septic arthritis, or other deep tissue involvement is present. The clinical course of the present patient differs from the treatment patterns generally reported for deeper *M. marinum* infections. Published clinical experience indicates that infections extending to tendon sheaths, joints, or bone are commonly treated with prolonged antimicrobial therapy, frequently combined with surgical intervention [1,2,5,23]. For example, deep hand and wrist infection has been successfully treated with surgical debridement followed by prolonged clarithromycin and ethambutol therapy [23]. These reports contrast with the present case, in which clinical resolution occurred after surgical drainage and only a short empirical course of ciprofloxacin, without subsequent targeted antimycobacterial treatment. This unusual outcome should be interpreted cautiously. It is possible that surgical source control played a major role, particularly because the infection appeared to remain localized and imaging did not demonstrate osteomyelitis or another deep organized focus. Local wound care and host-related factors may also have contributed. A contribution from ciprofloxacin cannot be excluded; however, the absence of antimicrobial susceptibility testing prevents assessment of whether the isolate was susceptible to this drug. Furthermore, clinical resolution cannot be equated with microbiological eradication. Follow-up mycobacterial cultures were not available, and the negative ZN staining only demonstrated the absence of microscopically detectable acid-fast bacilli in the sampled material. Therefore, the precise mechanism underlying recovery remains uncertain. Taken together, this case should not be interpreted as evidence that six days of ciprofloxacin is adequate therapy for deep *M. marinum* infection. Rather, it illustrates the potentially important role of surgical source control in selected localized infections and the limitations of inferring antimicrobial efficacy from a single favorable clinical outcome.

### 3.6. Limitations

Several limitations should be acknowledged. First, this is a single case, and therefore no causal inference can be made regarding the relationship between withdrawal of immunosuppressive therapy and clinical outcome. Second, antimicrobial susceptibility testing was not performed, so the susceptibility of the isolate to ciprofloxacin remains unknown. This limitation prevents any retrospective assessment of whether ciprofloxacin contributed to microbiological clearance. Third, follow-up mycobacterial cultures were not available. Therefore, the negative follow-up ZN staining should not be interpreted as definitive evidence of microbiological eradication. Fourth, although cross-reactivity between *M. marinum* and IGRA antigens is biologically plausible, the available data cannot establish that the patient’s positive QuantiFERON-TB Gold result was caused by *M. marinum* infection. Concomitant *M. tuberculosis* infection cannot be excluded solely on this basis [7,8]. Finally, the favorable outcome cannot be attributed to any single intervention. Surgical drainage, local wound management, the apparently limited anatomical extent of infection, host-related factors, and possibly the empirical antimicrobial exposure may all have contributed to the observed clinical resolution.

## 4. Conclusions

*M. marinum* infection should be considered in patients presenting with persistent, atypical, or treatment-refractory hand infections, particularly when there is a history of aquarium or aquatic exposure and underlying immunosuppression [1,2]. A detailed environmental history is an important component of the diagnostic assessment. Early surgical sampling, communication with the microbiology laboratory, incubation under appropriate temperature conditions, and species-level molecular identification can facilitate diagnosis and reduce diagnostic delay [1,2,20,25]. A positive QuantiFERON-TB Gold result should be interpreted in the appropriate clinical and epidemiological context. Although cross-reactivity with *M. marinum* is biologically plausible, the identification of *M. marinum* does not establish that the positive IGRA was caused by cross-reactivity. In transplant recipients, unsupervised modification of maintenance immunosuppression following an isolated positive IGRA should therefore be avoided.

The favorable outcome in this case after surgical drainage and a short empirical course of ciprofloxacin should not be interpreted as evidence supporting short-course ciprofloxacin monotherapy for deep *M. marinum* infection. The clinical resolution likely reflected a combination of surgical source control, local wound management, limited anatomical involvement, and host-related factors, while the contribution of ciprofloxacin remains uncertain. Because antimicrobial susceptibility testing and follow-up mycobacterial cultures were unavailable, neither antimicrobial efficacy nor definitive microbiological eradication could be established.

## Figures and Tables

**Figure 1:**
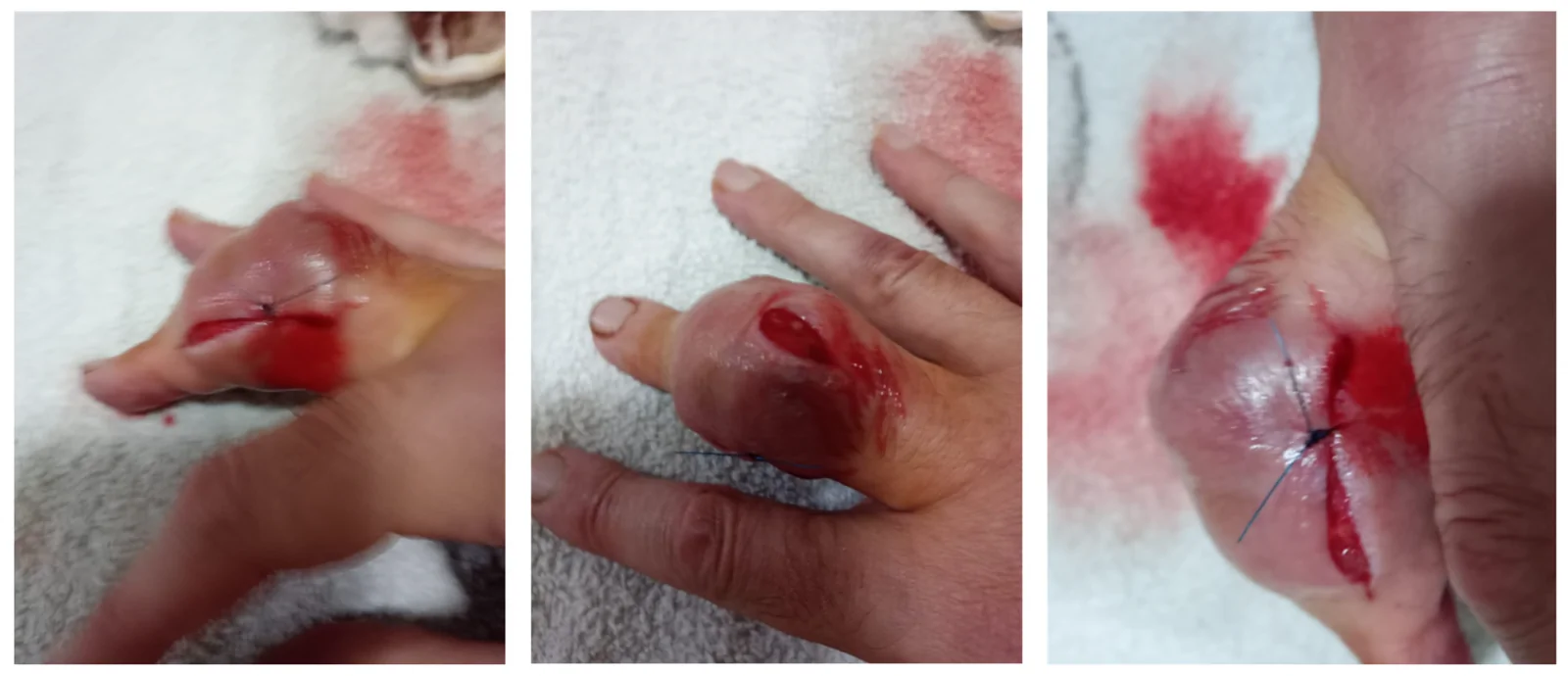
Clinical presentation of the right third finger showing marked fusiform swelling, erythema, and inflammatory changes before surgical intervention.

**Figure 2:**
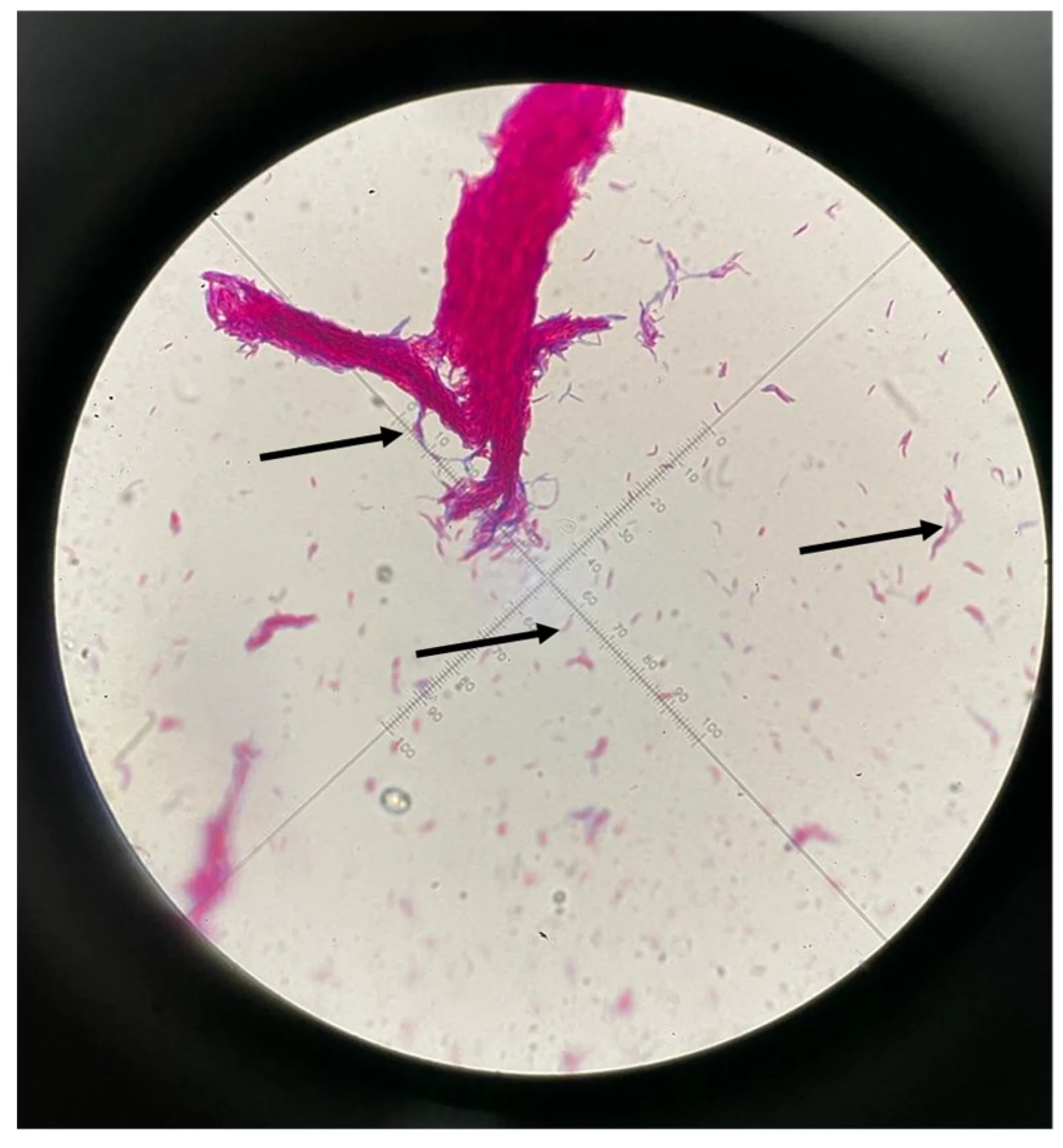
ZN staining of the surgical specimen demonstrating acid-fast bacilli. Arrowheads indicate representative acid-fast bacilli.

**Figure 3:**
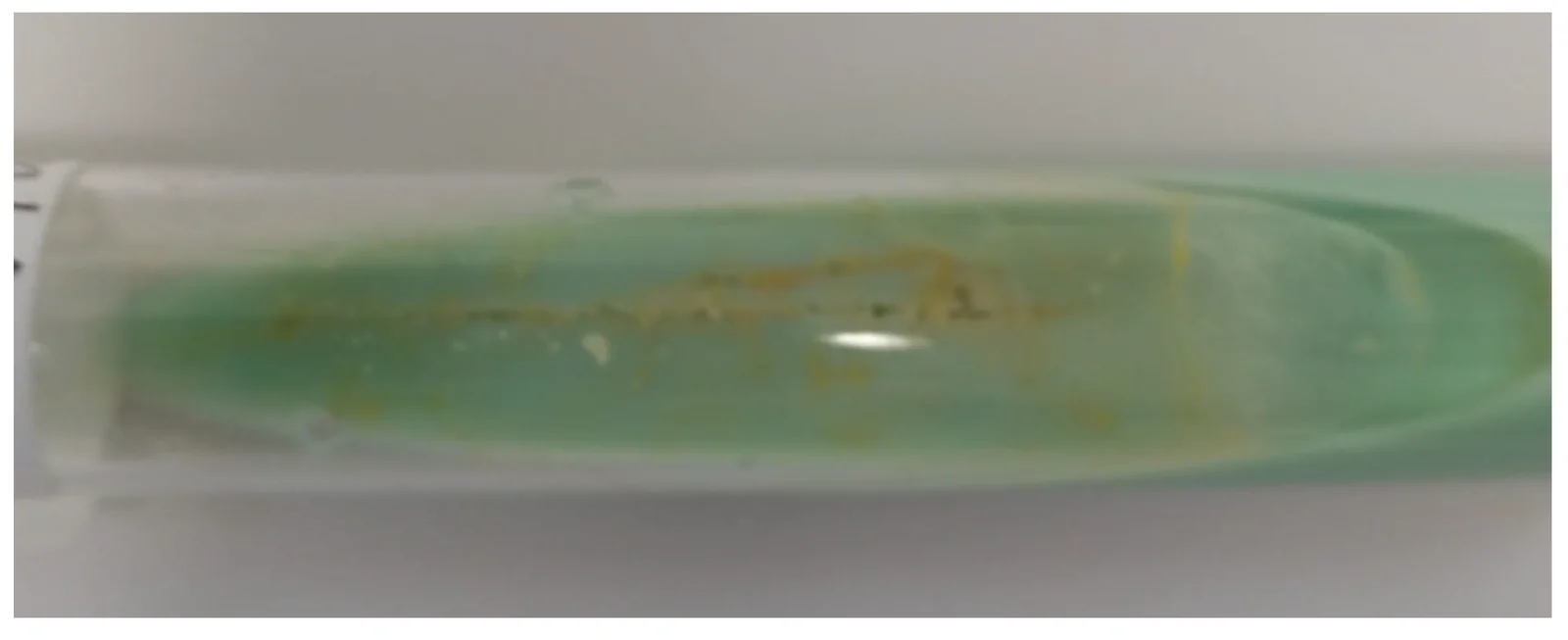
Photochromogenic colonies of *M. marinum* grown on Löwenstein–Jensen medium after approximately 40 days of incubation at 28–32 °C. Yellow–orange pigmentation developed following light exposure. Species identification was subsequently confirmed by molecular testing.

## Data Availability

The original contributions presented in this study are included in the article. Further inquiries can be directed to the corresponding author.

## Abbreviations

The following abbreviations are used in this manuscript:

IGRA/IGRAs	Interferon-Gamma Release Assay/Assays
*M.*	*Mycobacterium*
NTM	Nontuberculous mycobacteria
ZN	Ziehl–Neelsen

## References

[1] Canetti D., Riccardi N., Antonello R.M., Nozza S., Sotgiu G. (2022). *Mycobacterium marinum*: A brief update for clinical purposes. Eur. J. Intern. Med..

[2] Tsiolakkis G., Liontos A., Filippas-Ntekouan S., Matzaras R., Theodorou E., Vardas M., Vairaktari G., Nikopoulou A., Christaki E. (2023). *Mycobacterium marinum*: A Case-Based Narrative Review of Diagnosis and Management. Microorganisms.

[3] Moestrup P.G., Stilling M., Wejse C.M., Dahl V.N. (2023). *Mycobacterium marinum*: A Challenging Cause of Protracted Tenosynovitis. Antibiotics.

[4] Huang Y.Y., Li Q.S., Li Z.D., Sun A.H., Hu S.P. (2023). Rapid diagnosis of *Mycobacterium marinum* infection using targeted nanopore sequencing: A case report. Front. Cell Infect. Microbiol..

[5] Medel-Plaza M., Esteban J. (2023). Current treatment options for *Mycobacterium marinum* cutaneous infections. Expert Opin. Pharmacother..

[6] (2024). Kidney Disease: Improving Global Outcomes (KDIGO) CKD Work Group. KDIGO 2024 Clinical Practice Guideline for the Evaluation and Management of Chronic Kidney Disease. Kidney Int..

[7] Lewinsohn D.M., Leonard M.K., LoBue P.A., Cohn D.L., Daley C.L., Desmond E., Keane J., Lewinsohn D.A., Loeffler A.M., Mazurek G.H. (2017). Official American Thoracic Society/Infectious Diseases Society of America/Centers for Disease Control and Prevention Clinical Practice Guidelines: Diagnosis of Tuberculosis in Adults and Children. Clin. Infect. Dis..

[8] Lu J., Murugesan K., Senchyna F., Budvytiene I., Banaei N. (2023). Accuracy of QuantiFERON in active tuberculosis suspects with comorbidities and nontuberculous mycobacterial infection in Northern California. J. Clin. Microbiol..

[9] van Gelder T., Gelinck A., Meziyerh S., de Vries A.P.J., Moes D.J.A.R. (2025). Therapeutic drug monitoring of tacrolimus after kidney transplantation: Trough concentration or area under curve-based monitoring?. Br. J. Clin. Pharmacol..

[10] Rezahosseini O., Møller D.L., Knudsen A.D., Sørensen S.S., Perch M., Gustafsson F., Rasmussen A., Ostrowski S.R., Nielsen S.D. (2020). Use of T Cell Mediated Immune Functional Assays for Adjustment of Immunosuppressive or Anti-infective Agents in Solid Organ Transplant Recipients: A Systematic Review. Front. Immunol..

[11] Lewis F.M., Marsh B.J., von Reyn C.F. (2003). Fish tank exposure and cutaneous infections due to *Mycobacterium marinum*: Tuberculin skin testing, treatment, and prevention. Clin. Infect. Dis..

[12] Slany M., Jezek P., Bodnarova M. (2013). Fish tank granuloma caused by *Mycobacterium marinum* in two aquarists: Two case reports. Biomed. Res. Int..

[13] Slany M., Makovcova J., Jezek P., Bodnarova M., Pavlik I. (2014). Relative prevalence of *Mycobacterium marinum* in fish collected from aquaria and natural freshwaters in central Europe. J. Fish Dis..

[14] Kennedy C.D., Huang J.I., Hanel D.P. (2016). In Brief: Kanavel’s Signs and Pyogenic Flexor Tenosynovitis. Clin. Orthop. Relat. Res..

[15] Kennedy C.D., Lauder A.S., Pribaz J.R., Kennedy S.A. (2017). Differentiation Between Pyogenic Flexor Tenosynovitis and Other Finger Infections. Hand.

[16] Sproston N.R., Ashworth J.J. (2018). Role of C-Reactive Protein at Sites of Inflammation and Infection. Front. Immunol..

[17] Chapman T., Ilyas A.M. (2019). Pyogenic Flexor Tenosynovitis: Evaluation and Treatment Strategies. J. Hand Surg. Am..

[18] Park J.B., Seong S.H., Kwon D.I., Jang J.Y., Suh K.S., Jang M.S. (2020). *Mycobacterium marinum* Infection Spreading in a “Birds in Flocks” Pattern: All Caseating Granuloma is Not Tuberculosis. Acta Derm. Venereol..

[19] Travis W.D., Travis L.B., Roberts G.D., Su D.W., Weiland L.W. (1985). The histopathologic spectrum in *Mycobacterium marinum* infection. Arch. Pathol. Lab. Med..

[20] Aubry A., Mougari F., Reibel F., Cambau E. (2017). *Mycobacterium* *marinum*. Microbiol. Spectr..

[21] Johnson M.G., Stout J.E. (2015). Twenty-eight cases of *Mycobacterium marinum* infection: Retrospective case series and literature review. Infection.

[22] Hashish E., Merwad A., Elgaml S., Amer A., Kamal H., Elsadek A., Marei A., Sitohy M. (2018). *Mycobacterium marinum* infection in fish and man: Epidemiology, pathophysiology and management; a review. Vet. Q..

[23] Pop R., Estermann L., Schulthess B., Eberhard N. (2019). Deep infection with *Mycobacterium marinum*: Successful treatment of a frequently misdiagnosed disease. BMJ Case Rep..

[24] Ji C., Liu J., Mou Y., Cheng W., Hu W. (2023). Rapid Diagnosis of *Mycobacterium marinum* Infection on Both Hands using a Combination of Skin Trephination and Metagenomic Next-generation Sequencing. Acta Derm.-Venereol..

[25] Li L., Li M. (2022). Diagnosis of *Mycobacterium marinum* infection based on photochromogenicity: A case report. Rev. Inst. Med. Trop. Sao Paulo.

[26] Fishman J.A. (2017). Infection in Organ Transplantation. Am. J. Transplant..

[27] Baker R.J., Mark P.B., Patel R.K., Stevens K.K., Palmer N. (2017). Renal association clinical practice guideline in post-operative care in the kidney transplant recipient. BMC Nephrol..

